# Effect of Fertilization Regime of Common Wheat (*Triticum aestivum*) on Flour Quality and Shelf-Life of PDO Tuscan Bread

**DOI:** 10.3390/foods12142672

**Published:** 2023-07-11

**Authors:** Alessandro Bianchi, Chiara Sanmartin, Isabella Taglieri, Monica Macaluso, Francesca Venturi, Marco Napoli, Marco Mancini, Carolina Fabbri, Angela Zinnai

**Affiliations:** 1 Department of Agriculture, Food and Environment, University of Pisa, Via del Borghetto 80, 56124 Pisa, Italy; alessandro.bianchi@phd.unipi.it (A.B.); chiara.sanmartin@unipi.it (C.S.); isabella.taglieri@unipi.it (I.T.); monica.macaluso@unipi.it (M.M.); angela.zinnai@unipi.it (A.Z.); 2 Interdepartmental Research Centre “Nutraceuticals and Food for Health”, University of Pisa, Via del Borghetto 80, 56124 Pisa, Italy; 3 Department of Agriculture, Food, Environment and Forestry, University of Florence, Piazzale delle Cascine 18, 50144 Firenze, Italy; marco.napoli@unifi.it (M.N.); marco.mancini@unifi.it (M.M.); carolina.fabbri@unifi.it (C.F.)

**Keywords:** phosphorus, nitrogen, amylose, amylopectin, phytochemical, modified atmosphere packaging, sourdough, sensory analysis, breadmaking

## Abstract

The shelf-life of bread is influenced by flour components, such as starch, composed of amylose and amylopectin. The aim was to test the effect of different balances of N (45, 90, 135 kg/ha) and P (48, 96 kg/ha) fertilizers on the flour characteristics and consequently the shelf-life of PDO Tuscan bread, stored in different modified atmosphere packaging (Ar, N_2_, Air). The amylose and phytochemical compounds were increased by N and decreased by the addition of P, but excessive doses of N (135 kg/ha) had a negative effect on flour quality. In the bread, the study highlighted the tendency of N_2_ and Ar, as storage filler gases, to reduce water loss, slow down the staling process, and prolong shelf-life. However, the most significant influence on shelf-life was related to the different fertilizations of wheat. In fact, when N was present in equal dose to P (90/96 or 45/48 kg/ha) or slightly higher (90/48 kg/ha), the bread tended to last longer over time. Instead, when these ratios were unbalanced in favor of N (135/48 or 135/96 kg/ha) and in favor of P (45/96 kg/ha), the shelf-life decreased considerably.

## 1. Introduction

Wheat-based products are the most consumed foods all over the world, especially in the Mediterranean area [1]. In particular, the cultural tradition of breadmaking found its origins in Italy, where around 3.2 million tons of bread are produced and consumed per year [2]. The expected world population increase by 2050 and the economic globalization lay the foundations for reinvesting in bread production, looking not only to enhance quality and quantity production, but also the shelf-life of bread [3,4].

Based on the history and culture of the area, bread in Tuscany has developed into a staple food, like in many other Italian regions [5]. In 2013, Tuscan bread received the protected denomination of origin (PDO) by the Commission of the European Community [4]. The product specification requires specific flour and dough characteristics that are the sum of local varieties and environmental conditions of the origin area [5]. PDO Tuscan bread exhibits a considerably higher level of taste complexity than common commercial white bread because sourdough is used as a leavening agent [6,7], even without any salt added in the formulation. Additionally, the low pH and high levels of lactic and acetic acids in the crumb might also explain the longer shelf-life, which is mostly related to decreased mold spoilage and slowed staling process [1,4].

Bread composition is the result of a wide interaction of factors, including wheat genotypes, agronomic management, environmental conditions, flour composition, breadmaking conditions, and product storage [8,9]. The nutritional value and shelf-life of the final product are mainly influenced by starch composition [10] and in particular the amylose/amylopectin ratio [11,12]. Starch is present as granules and is the most important carbohydrate in wheat flour due to its water-absorbing capacity [13]. During bread storage, starch retrogradation is accompanied and driven by a complex process of moisture redistribution across the loaf, followed by moisture loss [14]. Furthermore, starch recrystallization can be significantly reduced also by the interaction between the gluten network and starch granules via hydrogen bonds [13,15]. As reported in the literature, moisture retention and water mobility play a vital role in the shelf-life of bread, especially during storage [1,4]. In this context, carbohydrates can slow down starch retrogradation, as they interfere in the interaction between water and starch [14,16].

Furthermore, macronutrients’ supply is one of the most important factors influencing wheat production, especially the application of nitrogen and phosphorus [17]. Both elements have to be managed following different application times and rates, and their application influences different aspects of crop production [18]. Nitrogen is an essential macroelement for wheat production, able to enhance grain yield, protein storage, starch composition, and, as a consequence, flour quality [19,20]. 

Nevertheless, the application of N is highly dependent on environmental conditions, such as soil structure and water availability [21,22,23]. Different authors [24,25,26,27,28] have shown that increasing N application in wheat is able to increase protein concentration, in particular, a different modality of N application modifies the protein composition (gliadin and glutenin proportions) as well as the quality of cooking [29]. Zhou et al. (2020) [30] suggest that increasing N from 0 to 100 kg/ha improves amylose and amylopectin contents, but that excess nitrogen decreases starch content. Xue et al. (2016) [31] found that splitting N application influences the composition of the grain, influencing the wheat flour quality, and that delaying the N supply is able to influence breadmaking quality, favoring protein build-up.

Furthermore, phosphorus supply is important to ensuring the production of energy from photosynthesis and transportation of carbohydrates, root growth, and increasing yield [32]. However, soils are usually P deficient due to rapid element immobilization, highly dependent on soil pH and organic matter content [33]. Zhang et al. (2017) [34] found that applying P to wheat from 0 to 400 kg/ha increases yield by up to 50 kg ha, but the protein concentration in grain and flour decreases. Indeed, Guerrini et al. (2020) [27] showed that P fertilization is able to increase starch content, but significantly reduces the ratio of amylose to amylopectin.

To strengthen the impact of the final product on the market and increase the sustainability of the bread supply chain, knowing the effect of management practices on wheat varieties and dough quality and quantity characteristics would be useful. 

This is relevant for storage bread, as refrigerating storage of freshly baked bread is not applicable because its texture and taste are negatively affected by low temperatures [35]. To preserve both the sensory qualities and nutritional content of PDO Tuscan bread while extending its shelf-life without using preservatives, whose use is prohibited by the traditional recipe, proper modified atmosphere packaging (MAP) appears to be the most effective strategy [36,37].

Bianchi et al. (2022) [4] recently showed that the use of 100% Ar or 100% N_2_ for the MAP can be the best solution to preserve PDO Tuscan bread and a good compromise from a chemical–physical point of view, but above all, on a sensory level, because the use of CO_2_ is not recommended with the high level of acidity of this type of sourdough bread [7].

For this reason, the aim of this work was to evaluate how different nitrogen and phosphorus rates applied to the wheat genotypes allowed for PDO Tuscan bread production influenced the flour features and consequently the shelf-life of the bread stored in modified atmosphere packaging (Ar, N_2_, air).

## 2. Materials and Methods

### 2.1. Experimental Field

Field experiments were carried out for two consecutive growing seasons from September 2018 to August 2020 under rainfed conditions in Pienza, Tuscany, Italy (42.986569° N, 11.763888° E, 330 m a.s.l.). The 0–0.3 m soil layer was silty clay loam (Aquic Haplustepts, fine, mixed, mesic), sub-alkaline (pH 8.1), and contained 13.8 g/kg of total organic carbon, 1.2 g/kg of total nitrogen, and 7.6 mg/kg of available phosphorus.

**Figure 1:**
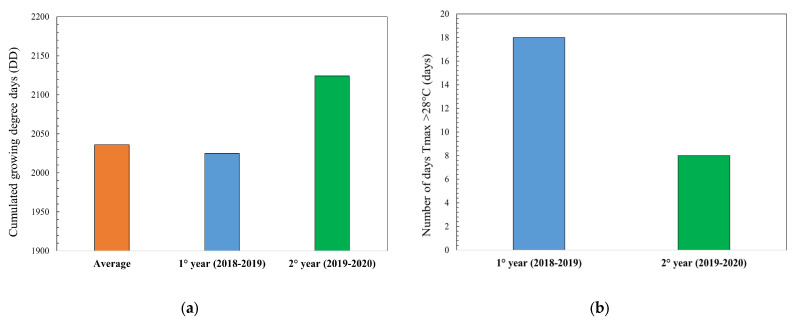
Climatic parameters in the two years during the growing season of November–June: (**a**) cumulated growing degree days (DD); (**b**) number of days with maximum temperatures above 28 °C.

The treatments consisted of factorial combinations of two phosphorus (P) levels (48 and 96 kg/ha), three nitrogen (N) levels (45, 90 and 135 kg N/ha), and four common wheat varieties (*Triticum aestivum*) allowed in the mix for PDO Tuscan bread production, for a total of 24 treatments. The four bread varieties comprised three registered dwarf varieties (namely Panda, Bolero, and Bologna) and one old, non-dwarf landraces (namely Verna). The experiment field was arranged in a strip-plot design with three replicate blocks per year (Figure S1). Egyptian clover (*Trifolium alexandrinum*, L.) was the previous crop in both growing seasons. In both seasons, the fields were plowed and then disk harrowed in late October. Then, phosphorous (triple superphosphate; P_2_O_5_: 46%) was distributed homogeneously on the soil surface and incorporated by disk harrowing at a 5 cm depth. Common wheat seeds were sown in December 2018 and 2019 with an inter row distance of 13 cm. The total dose of nitrogen was distributed in three applications: 20% at sowing by broadcasting urea (N: 46%), 50% by broadcasting ammonium nitrate (N: 26%) at tillering, and 50% by broadcasting urea (N: 46%) at stem elongation. Common wheat from each treatment was harvested separately using a plot combine-harvester equipped with Trimble GPS sensors. For each treatment, 5 kg of harvested wheat kernel were sampled for preparing the flour mix to be used for the analyses and the breadmaking trials. Weather conditions were monitored by consulting data acquired from a weather station located in the field where the experiment was being conducted. During the growing and production seasons (November–June), cumulative rainfall (mm) and cumulative growing degree days (DD) were calculated and analyzed. The cumulative degree days value was calculated daily as the difference between the average daily temperature and the base temperature considered useful for growth and development of wheat. For the characterization of the entire crop cycle, a thermal threshold of 4 °C was taken into consideration as reported by Saiyed et al. (2009) [38]. The analysis of the thermometric trend showed that compared to an average thermal of 2036 DD, the first year of tests (2018–2019) was substantially in line, while in the second year (2019–2020), there was a positive anomaly of 76 DD mainly due to a mild winter (Figure 1a). Conversely, in the maturation phase, there were more days with maximum temperatures above 28 °C in the 2018–2019 season, with June 2019 recording 18 hot days, compared to 8 days in the second year (Figure 1b).

**Figure 2:**
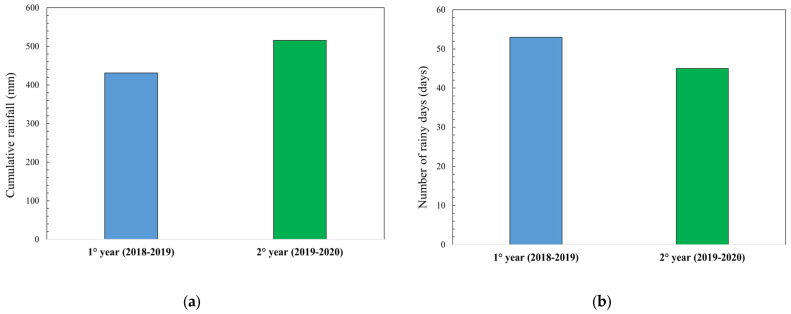
Climatic parameters in the two years during the growing season of November–June: (**a**) cumulated rainfall (mm); (**b**) number of rainy days.

Rainfall during the 2018–2019 production season was 431 mm, and 515 mm in the 2019–2020 season (Figure 2a). Analyzing the distribution of rainfall, the first year showed a much higher number of rainy days than the second did (Figure 2b). However, a monthly analysis showed a slight winter drought in the first year of trials (2018–2019) and excessive rainfall in June 2020 that affected the final stage of wheat ripening. The final stage of cereal ripening plays a key role in starch accumulation and protein translocation. In fact, very high temperatures in June are often the cause of a sudden senescence of the plant with interruption of starch synthesis and accumulation [38].

### 2.2. Characterizzation of Flours

According to the specifications for PDO Tuscan bread production, the flour was obtained by a mix of four varieties of common wheat divided for the six combination of N/P (kg/ha) treatments (M1 = 45/48, M2 = 45/96, M3 = 90/48, M4 = 90/96, M5 = 135/48, M6 = 135/96) produced in the two years (2019 and 2020 crop seasons). 

A commercial mill (Industry-Combi, Waldner Biotech, Lienz, Austria) was used for the milling process at the Department of Agricultural, Food, and Environment (DAFE) at the University of Pisa.

The chemical composition and the technological features of flours were determined as previously reported [4] according to the methods accepted by the International Organization for Standardization (ISO) and by the Association of Official Analytical Chemists International (AOAC): humidity (ISO 712:2009); ashes (ISO 2171:2007); proteins (ISO 20483:2013); total fats (ISO 11085:2015); falling number (ISO 3093:2009); wet gluten and gluten index (ISO 21415-2:2015); dry gluten (ISO 21415-3:2006); total dietary fiber (AOAC 2011.25-2012); sugars (AOAC 982.14-1983); amylose and amylopectin (ISO 6647-1:2020); total starch (AOAC 996.11-2005); Chopin alveogram (W, P/L, P, L, G) (ISO 27971:2015); Brabender farinogram (water absorption corrected to 14% humidity, dough time, stability, softening degree (E10: degree of softening after 10 min; E(ICC): softening degree 12 min, after max), and FQN: number of farinographic quality) (ISO 5530-1:2013).

Moreover, the flour was characterized from a phytochemical point of view (total polyphenols, total flavonoids, and anti-radical activity). In particular, an 80% methanol solution was used to perform a solid/liquid extraction (ratio 1/20 *w*/*v*) from 0.5 g of a fresh flour sample, sonicating the mixture for 30 min. All the extracts were subsequently centrifuged (15 min, 3500 rpm), filtered on a syringe filter (0.45 μm), and stored at 4 °C for the immediate analysis. 

The Folin–Ciocalteu colorimetric method was applied for total polyphenols spectrophotometry as previously reported [39], expressing the results as milligrams of gallic acid equivalents (GAE) per kilogram of dry matter (dm). The total flavonoids were estimated according to the procedure reported by Bianchi et al. (2023) [40], comparing the measures to a standard curve of catechin, and the results were reported as milligrams of catechin equivalents (CE) per kilogram of dm. Using the free radical methods (FRAP [40], ABTS [41], and DPPH [40]), the anti-radical activity of the extracts was determined. According to different standard curves of Trolox (range: 0–2.0 mM for the FRAP H, 0.2–1.5 mM range for ABTS, and 0–200 µmol/L for the DPP), the results were expressed as micromoles of Trolox equivalents (TE) per gram of dm.

### 2.3. Breadmaking Process

The Consortium for the protection of PDO Tuscan Bread procured the sourdough used in the study. Consecutive back slopping was used for preserving the sourdough in order to maintain its acidifying and leavening properties [42]. Breadmaking was carried out from a pre-ferment leavening agent, according to the method of “biga”. According to the production specification for PDO Tuscan bread, for all the experimental runs, sourdough biga was prepared by mixing a strong wheat flour type 0 (56% *w*/*w*) and sterile water (33% *w*/*w*), and (11% *w*/*w*) then leaving to ferment for 18 h at 20 °C. For each experimental run, the specific formulation was produced with 32% of water, 16% of leavening agent (biga), and 52% of flour selected among the six different treatments (M1–M6) described in Section 2.2. The first leavening lasted for 90 min at 26 ± 1 °C, then, the dough was broken and shaped and left for a further 2.5 h at 35 ± 1 °C (second leavening). Finally, the loaves were baked at 220 °C for 45 min.

### 2.4. Bread Shelf-Life Assessment

After baking, the bread loaves were cooled for 2 h at room temperature (23 ± 1 °C), then sliced with an automatic slicing machine to a 20 mm thickness. Each slice was packed individually in plastic bags (two plastic layers, outer nylon layer, Food Saver, Moncalieri, Torino, Italy), by an industrial packaging machine (Lavezzini 450 GAS, Fiorenzuola d’Arda, Piacenza, Italy). Twenty loaves (1 kg each) for the six treatments were prepared, 500 slices each were packed separately in three different MAPs (Ar (100%), N_2_ (100%), air (100%)). Each pack was stored at a controlled temperature (23 °C) during the whole observation period.

In this study, the samples are represented by bread cut into slices, not by the whole loaf, as the result is a much larger exposed surface that determines a more rapid evolution of the parameters. The choice of slices was made in order to be able to study the process in an accelerated-shelf-life way, as reported in a previous study [4].

Four sliced samples of each storage condition were opened each day and subjected to the following analysis in order to evaluate the bread shelf-life as a function of the flours and storage conditions.

Slices were weighed daily for each experimental run to determine the weight loss brought on by the evaporation of water from the slices during storage; the value was shown as a percentage decrease from the starting value.

Water activity (a_w_) was measured by a HygroPalm HP23-AW-A equipment (Rotronic AG, Bassersdorf, Switzerland). The results were calculated as a percentage reduction in water activity compared to the initial value.

As reported by Bianchi et al. (2022) [4], the softness of the crumb was determined by a penetrometer PNR-12 (Anton Paar, Rivoli, Italy), and the results were expressed as a percentage reduction in softness compared to the initial value.

All the samples were checked daily for the presence of mold; each experimental run was stopped when 3% of the samples showed mold spoilage.

Finally, the sensory profiles of the bread samples were evaluated by a panel of eight trained judges (aged between 23 and 60 years) of the Department of Agriculture, Food, and Environment Sciences of the University of Pisa. The tasting was carried out according to a previously developed protocol [43], and the overall hedonic index of bread was calculated as reported by Bianchi et al. (2022) [4]. The research obtained the approval of the Ethics Committee of the University of Pisa (protocol no. 0088081/2020).

### 2.5. Statistical Analysis

All the physical chemical parameters were evaluated in quadruplicate.

One-way ANOVA (CoStat, Version 6.451, CoHort Software, Pacific Grove, CA, USA) was used to assess the significance of the difference between the samples, and Tukey’s HSD test (*p* ≤ 0.05) was used for the separation of the samples.

On the parameters of flour quality, two-way ANOVA was also used to determine the effect of the two different factors (year or treatments or the combination) on the parameters of flour quality.

The trend of the shelf-life parameters over time (decrease of weight, water activity, and softness) and the linear regression were elaborated with the JMP software package version 17 (SAS Institute, Cary, NC, USA).

Big Sensory Soft 2.0 software (ver. 2018) analyzed the findings of the sensory analysis. Panelists and samples were the factors in the two-way ANOVA used to assess the sensory data [4].

## 3. Results and Discussion

### 3.1. Flour Quality Characterization

Among the analyses carried out on the various types of flours related to two successive years (2019 and 2020), the most significant parameters related to further bread spoilage attitude, mainly related to the staling process and mold development, are reported below and further discussed (Table 1 and Table 2). All data showed almost the same trend as a function of fertilization rate, regardless of the crop season analyzed.

Further, as reported in Table S1, the most significant differences among the flour samples were determined by treatment, followed by year crop season, while only a few parameters were significantly affected by the combination “treatment × year”.

According to Rekowski et al. (2019) [25], higher protein content in flour determines a reduction in the loss of free water during staling, because protein’s water retention power allows it to gradually counteract the starch recrystallization process. A greater amount of protein and starch were induced by the highest nitrogen fertilization rate (flours M5 and M6), followed by that with M3 and M4 flours, while the lowest values were reported for M1 and M2 flours. 

As the sugar content increases, the gelatinization temperature increases too, while the rate of the starch retrogradation decreases [16]. In the experimental conditions, flours M1, M3, and M4 had the highest sum of total sugars compared to that of the other flours. The concentration of gluten, both dry and wet, is fundamental to evaluating the breadmaking properties of flour [10]. The higher its value, the more the flour is suitable for this purpose. M5 and M6 flours showed the highest gluten concentration among others, while M2 flour showed the lowest one, thus suggesting that the increase in N fertilization had a positive effect on this parameter [30].

According to Schirmer et al. (2013) [11], amylose/amylopectin content is directly proportional to starch retrogradation rate, as amylose tends to recrystallize much faster than amylopectin. Looking at the results obtained in the two years (Table 1 and Table 2), it is clear that the flours M1, M3, and M4 contained statistically significantly lower amylose content than the others and therefore could show a reduced tendency of staling [10,11].

Furthermore, phenolic compounds have an inhibitory activity towards the development of microorganisms; therefore, a high concentration of them determines a delayed appearance of fungal bodies on the bread. In addition, as reported in [40], the combination with sourdough leavening allows for their increase in the produced bread, thanks to the ability to increased availability and bio-accessibility of this phytochemical compound. Flours M1, M3, and M4 had a statistically higher quantity of phenolic compounds (both total polyphenols and total flavonoids) and higher antioxidant power compared with those of the other flours (Table 1 and Table 2). 

Taken together, the data showed that the six flour mixes could be divided into two main groups showing both a different chemical composition and potentially different technological features mainly related to bread spoilage, with flours M5 and M6 being clearly different from flours M1 and M2, regardless of the crop season analyzed.

### 3.2. Trend of Weight Loss, Water Activity, and Softness

Water migration consists of the redistribution of free water molecules due to the moisture gradient between different areas of the product, mainly from the crumb to the crust, and this migration contributes strongly to the phenomenon of staling. During storage, the water initially included in the gel fraction of starch is gradually released because of the starch recrystallization process, thus driving further crumb softness loss typical of bread staling. 

To evaluate the shelf-life trend during the storage period as a function of storage atmosphere composition, the linear regression of weight loss (Figure S2a,b) together with the water activity decrease (Figure S3a,b) was calculated. The slopes of the regression lines and R^2^ are reported in Table 3 for the two-year observation period.

According to what was reported about the flour’s features as a function of fertilization rate, both weight loss and water activity decrease showed the same trend in all the experimental conditions tested, regardless of the crop season analyzed.

As expected [4], given the flour mix, significantly faster water loss was detected when the bread was stored in air (Table 3).

**Figure 3:**
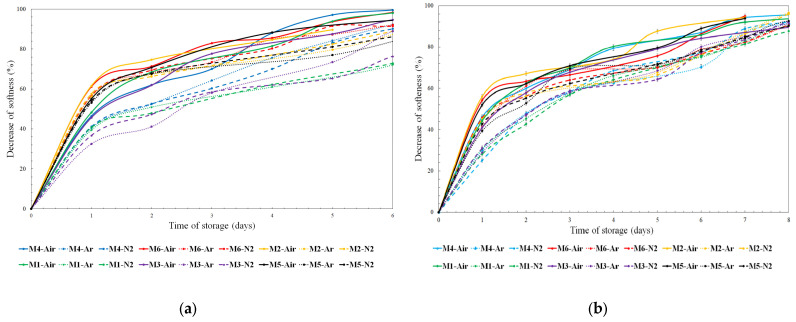
Trend of the decrease of softness (%) as a function of storage days: (**a**) year 2019; (**b**) year 2020.

Furthermore, given the storage atmosphere, the flour composition represented the main factor in determining the crumb softness reduction rate (Figure 3a,b), with a lower one measured for breads produced with flours M1, M3, and M4, while flours M2, M5, and M6 significantly reduced the bread’s shelf-life. Furthermore, the technological characteristics of the flour induced by the different fertilization regimes appeared to be more important than the storage atmosphere in determining the bread’s shelf-life. Indeed, breads produced with flours M1, M3, and M4 stored in air showed the same trend as when breads produced with flours M2, M5, and M6 were stored in an inert atmosphere (100% Ar or 100% N_2_).

### 3.3. Mold Appearance

Molds determine the decay of the organoleptic quality that derives from the production of off-flavors in terms of alcohols and esters, which cause unpleasant hints and the appearance of spots with an abnormal color on the surface of the product.

**Figure 4:**
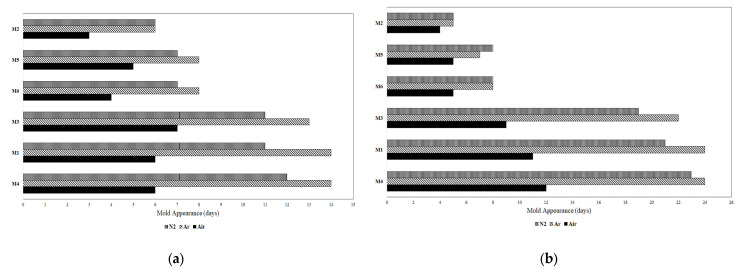
Mold appearance on the surface of bread: (**a**) year 2019; (**b**) year 2020.

As shown in Figure 4a,b, flours M1, M3, and M4 significantly delayed mold development, thus improving the bread’s shelf-life regardless of the storage atmosphere or the crop season. These results can be explained well by the high phenolic content detected in flours M1, M3, and M4. Among the different gas compositions of storage atmospheres, as expected, air induced the fastest mold development.

### 3.4. Sensory Evaluation of Bread

Breads produced with the different flours were assessed before packaging to determine their sensory profile at t = 0 (Figure S4a,b), and flours M1, M3, and M4 produced the breads with the best sensory profiles.

During the whole observation period, further panel tests were performed daily on the breads stored in different gas atmospheres until the first molds were observed on the slice surface of the sample stored in the worst conditions.

As expected, a decrease in overall pleasantness during storage was observed due to the staling phenomenon, where the breads produced with flours M1, M3, and M4 were still acceptable (HI > 6) at the end of the trials regardless of the storage atmosphere, thus confirming the main effect of flour composition discussed above. 

**Figure 5:**
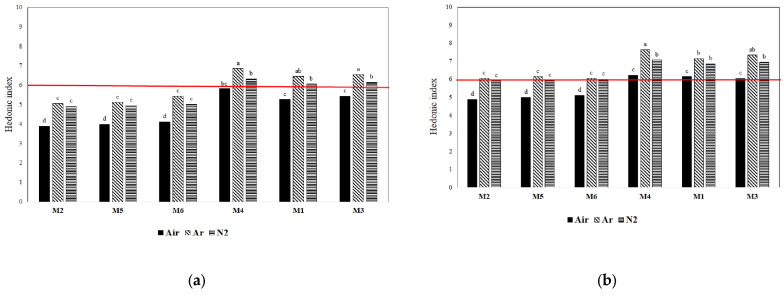
Hedonic index (HI) of the different breads in the three systems of MAP at the end of the storage period: (**a**) year 2019; (**b**) year 2020. The red line indicates the HI reference limit of shelf-life. Different lowercase letters indicate significant differences at *p* < 0.05.

When gas composition was assumed as the main effect (Figure 5a,b), the best sensory profile was observed for breads stored in 100% Ar, probably due to its superior antioxidant power that better preserved aromatic molecules.

## 4. Conclusions

The study confirmed that N_2_ and Ar could reduce water loss, slow down the staling process, and allowed the bread to not only last longer but also to better maintain its initial characteristics, compared to those with air. However, a more important effect is linked to differences in fertilizations of the wheat flour. In fact, the breads using the flours M1, M3, and M4 in air had the same results as those of the flours M2, M5, and M6 in a protective atmosphere of Ar and N_2_.

During two different crop seasons with different climatic behaviors, the main parameters related to flour quality and composition were mainly affected by the fertilization approach, followed by crop season, while only a few parameters were significantly affected by the combination “treatment × year”.

In fact, in N/P the fertilization, when N was present in a dose equal to that of P (90/96, 45/48 and 90/48 kg/ha) or in a higher but not excessive dose (90/48 kg/ha), the bread obtained tended to have better characteristics and to last longer over time. When the N/P ratios were unbalanced in favor of nitrogen (135/48 and 135/96 kg/ha) or in favor of phosphorus (45/96 kg/ha), the shelf-life and also the chemical–physical and sensory characteristics strongly decreased. 

In conclusion, the flours obtained with less abundant fertilizations of N and P had better results, representing an opportunity for producers to save by administering lower doses of fertilizer. At the same time, there would be an environmental benefit, as less energy and chemical inputs would be used.

## Figures and Tables

**Table 1:** Chemical and technological parameters of the six flours used in the breadmaking trial (year 2019).

		Year 2019						
Parameters	Units	^1^	M1	M2	M3	M4	M5	M6
Chemical								
Humidity	% *w*/*w*	ns	11.13	10.92	11.28	11.00	10.05	10.82
Ashes	% *w*/*w*	ns	1.24	1.17	1.26	1.08	1.09	1.13
Proteins	% *w*/*w*	**	13.34 ^c^	13.42 ^c^	13.69 ^b^	13.98 ^ab^	14.26 ^a^	14.51 ^a^
Total fats	% *w*/*w*	ns	2.05	2.18	2.14	1.98	2.06	2.15
Total dietary fiber	% *w*/*w*	**	5.02 ^c^	6.83 ^a^	5.38 ^c^	5.31 ^c^	6.12 ^b^	6.21 ^b^
Sucrose	% *w*/*w*	*	0.79 ^ab^	0.68 ^c^	0.87 ^a^	0.86 ^a^	0.71 ^bc^	0.72 ^bc^
Glucose	% *w*/*w*	*	0.293 ^ab^	0.22 ^b^	0.30 ^a^	0.33 ^a^	0.22 ^b^	0.24 ^b^
Fructose	% *w*/*w*	ns	0.10	0.08	0.12	0.10	0.09	0.11
Maltose	% *w*/*w*	***	5.02 ^ab^	4.42 ^d^	5.21 ^a^	5.28 ^a^	4.90 ^b^	4.70 ^c^
Wet gluten	% *w*/*w*	**	38.72 ^b^	36.12 ^c^	38.65 ^b^	38.14 ^b^	41.22 ^a^	41.86 ^a^
Dry gluten	% *w*/*w*	**	11.51 ^c^	10.43 ^d^	11.54 ^c^	11.70 ^c^	11.94 ^b^	12.84 ^a^
Gluten index	% *w*/*w*	**	67.64 ^c^	63.21 ^d^	70.74 ^b^	67.33 ^c^	69.12 ^b^	71.92 ^a^
Total Starch	% *w*/*w*	**	83.64 ^cd^	83.02 ^d^	84.85 ^ab^	84.03 ^b^	85.09 ^a^	85.71 ^a^
Amylose	% *w*/*w*	***	22.82 ^c^	24.64 ^b^	22.64 ^c^	22.72 ^c^	25.73 ^a^	25.42 ^a^
Amylopectin	% *w*/*w*	***	77.21 ^c^	75.44 ^b^	77.48 ^c^	77.31 ^c^	74.32 ^a^	74.68 ^a^
Falling number	seconds	ns	303	298	300	310	312	316
Total polyphenol	mg GAE/kg dm	***	635 ^a^	449 ^c^	617 ^b^	642 ^a^	440 ^c^	416 ^d^
Total flavonoids	mg CE/kg dm	**	55.82 ^ab^	46.43 ^c^	53.45 ^b^	57.42 ^a^	40.44 ^d^	48.42 ^c^
ABTS	μmol TE/g dm	***	0.79 ^a^	0.53 ^c^	0.68 ^b^	0.82 ^a^	0.50 ^c^	0.41 ^d^
DPPH	μmol TE/g dm	**	0.40 ^ab^	0.32 ^c^	0.38 ^b^	0.45 ^a^	0.31 ^c^	0.35 ^c^
FRAP	μmol TE/g dm	***	0.79 ^a^	0.56 ^c^	0.81 ^a^	0.84 ^a^	0.55 ^c^	0.48 ^d^
**Technological**								
W	10^−4^ joules	*	229 ^c^	248 ^bc^	237 ^b^	240 ^b^	258 ^a^	262 ^a^
P/L		*	1.62 ^b^	1.92 ^a^	1.54 ^b^	1.67 ^b^	1.98 ^a^	2.06 ^a^
P	mm	ns	106	112	105	112	113	114
L	mm	*	48 ^a^	40 ^b^	49 ^a^	48 ^a^	41 ^b^	39 ^b^
G		*	13.5 ^a^	12.6 ^b^	13.6 ^a^	13.4 ^a^	12.7 ^b^	12.7 ^b^
Water absorption	%	**	67.4 ^c^	73.1 ^a^	67.4 ^c^	67.2 ^c^	72.0 ^b^	71.5 ^b^
Dough time	Minutes	ns	5.6	5.2	5.8	5.3	5.5	5.3
Stability	Minutes	ns	5.1	4.6	4.9	4.8	4.6	4.9
E10	UF	ns	41	50	52	50	48	42
E(ICC)	UF	ns	74	72	74	80	79	81
FQN		ns	75	64	70	72	68	65

^1^ Significance level: *** *p* ≤ 0.001; ** *p* ≤ 0.01; * *p* ≤ 0.05; ns = not significant (*p* > 0.05). In the same row, different letters indicate significant differences among samples.

**Table 2:** Chemical and technological parameters of the six flours used in the breadmaking trial (year 2020).

		Year 2020						
Parameters	Units	^1^	M1	M2	M3	M4	M5	M6
Chemical								
Humidity	% *w*/*w*	ns	11.08	11.22	10.85	10.90	11.05	11.22
Ashes	% *w*/*w*	ns	1.34	1.37	1.36	1.37	1.30	1.33
Proteins	% *w*/*w*	**	12.24 ^c^	12.52 ^bc^	12.64 ^b^	12.90 ^b^	13.46 ^a^	13.50 ^a^
Total fats	% *w*/*w*	ns	2.45	2.52	2.44	2.50	2.46	2.45
Total dietary fiber	% *w*/*w*	*	5.82 ^d^	8.83 ^a^	6.58 ^c^	6.70 ^bc^	7.32 ^b^	7.41 ^b^
Sucrose	% *w*/*w*	*	0.94 ^ab^	0.80 ^c^	0.97 ^a^	0.96 ^a^	0.91 ^b^	0.89 ^b^
Glucose	% *w*/*w*	**	0.43 ^ab^	0.31 ^c^	0.46 ^a^	0.45 ^a^	0.39 ^b^	0.38 ^b^
Fructose	% *w*/*w*	ns	0.12	0.13	0.11	0.14	0.13	0.13
Maltose	% *w*/*w*	**	7.22 ^a^	6.52 ^c^	7.21 ^a^	7.28 ^a^	6.75 ^b^	6.70 ^b^
Wet gluten	% *w*/*w*	**	39.72 ^c^	36.22 ^d^	39.65 ^c^	39.14 ^c^	41.22 ^b^	42.86 ^a^
Dry gluten	% *w*/*w*	**	12.51 ^c^	11.63 ^d^	12.54 ^c^	12.90 ^c^	12.94 ^b^	13.84 ^a^
Gluten index	% *w*/*w*	*	67.60 ^d^	69.21 ^cd^	73.71 ^b^	75.32 ^a^	72.14 ^bc^	73.92 ^b^
Total Starch	% *w*/*w*	**	86.29 ^b^	85.03 ^c^	86.65 ^b^	86.71 ^b^	88.54 ^a^	88.32 ^a^
Amylose	% *w*/*w*	***	20.72 ^b^	23.64 ^a^	21.04 ^b^	20.82 ^b^	23.73 ^a^	23.42 ^a^
Amylopectin	% *w*/*w*	***	79.31 ^b^	76.44 ^a^	79.03 ^b^	79.22 ^b^	76.32 ^a^	76.68 ^a^
Falling number	seconds	ns	333	332	340	327	318	326
Total polyphenol	mg GAE/kg dm	**	835 ^a^	749 ^b^	827 ^a^	842 ^a^	719 ^c^	716 ^c^
Total flavonoids	mg CE/kg dm	***	75.82 ^a^	63.43 ^c^	73.45 ^b^	77.42 ^a^	60.44 ^d^	58.42 ^e^
ABTS	μmol TE/g dm	**	1.15 ^ab^	0.83 ^c^	1.09 ^b^	1.23 ^a^	0.79 ^c^	0.66 ^d^
DPPH	μmol TE/g dm	**	0.70 ^ab^	0.52 ^c^	0.65 ^b^	0.75 ^a^	0.51 ^cd^	0.45 ^d^
FRAP	μmol TE/g dm	***	1.50 ^a^	1.22 ^c^	1.41 ^b^	1.54 ^a^	1.16 ^c^	1.01 ^d^
**Technological**								
W	10^−4^ joules	*	245 ^d^	262 ^bc^	258 ^c^	259 ^c^	273 ^ab^	282 ^a^
P/L		*	2.62 ^b^	2.92 ^a^	2.54 ^b^	2.67 ^b^	2.98 ^a^	3.06 ^a^
P	mm	ns	156	162	150	152	160	160
L	mm	*	63 ^a^	56 ^b^	61 ^a^	68 ^a^	51 ^b^	56 ^b^
G		*	15.5 ^a^	14.1 ^b^	15.6 ^a^	15.4 ^a^	14.7 ^b^	14.8 ^ab^
Water absorption	%	**	68.4 ^b^	69.7 ^a^	68.4 ^b^	68.2 ^b^	69.9 ^a^	69.5 ^a^
Dough time	Minutes	ns	4.6	4.2	4.8	4.3	4.5	4.3
Stability	Minutes	ns	6.2	5.5	5.9	5.4	6.4	6.2
E10	UF	ns	46	59	48	46	58	52
E(ICC)	UF	ns	81	92	81	90	87	87
FQN		ns	85	73	80	81	74	75

^1^ Significance level: *** *p* ≤ 0.001; ** *p* ≤ 0.01; * *p* ≤ 0.05; ns = not significant (*p* > 0.05). In the same row, different letters indicate significant differences among samples.

**Table 3:** Slope and the coefficient of determination (R^2^) for the linear regression of the decrease of weight and the decrease of water activity (a_w_) in the two years (2019 and 2020).

Sample	Year 2019				Year 2020			
	Slope (Weight)	R^2^	Slope (a_w_)	R^2^	Slope (Weight)	R^2^	Slope (a_w_)	R^2^
M1-Air	0.448 ^c^	0.987	0.361 ^c^	0.972	0.473 ^b^	0.986	0.261 ^b^	0.962
M1-Ar	0.333 ^d^	0.993	0.236 ^d^	0.996	0.321 ^c^	0.984	0.135 ^c^	0.973
M1-N_2_	0.344 ^d^	0.995	0.255 ^d^	0.994	0.352 ^c^	0.986	0.155 ^c^	0.968
M2-Air	0.643 ^a^	0.970	0.509 ^a^	0.964	0.571 ^a^	0.990	0.376 ^a^	0.960
M2-Ar	0.466 ^bc^	0.980	0.380 ^bc^	0.982	0.478 ^b^	0.991	0.303 ^b^	0.976
M2-N_2_	0.500 ^b^	0.979	0.420 ^b^	0.974	0.473 ^b^	0.986	0.306 ^b^	0.959
M3-Air	0.441 ^c^	0.988	0.359 ^c^	0.984	0.445 ^b^	0.992	0.265 ^b^	0.982
M3-Ar	0.329 ^d^	0.995	0.236 ^d^	0.994	0.347 ^c^	0.998	0.156 ^c^	0.978
M3-N_2_	0.341 ^d^	0.978	0.250 ^d^	0.985	0.341 ^c^	0.983	0.158 ^c^	0.968
M4-Air	0.440 ^c^	0.991	0.353 ^c^	0.991	0.455 ^b^	0.992	0.273 ^b^	0.963
M4-Ar	0.321 ^d^	0.994	0.238 ^d^	0.993	0.336 ^c^	0.985	0.132 ^c^	0.972
M4-N_2_	0.327 ^d^	0.997	0.249 ^d^	0.998	0.345 ^c^	0.993	0.149 ^c^	0.965
M5-Air	0.625 ^a^	0.969	0.506 ^a^	0.967	0.548 ^a^	0.988	0.388 ^a^	0.966
M5-Ar	0.534 ^b^	0.991	0.388 ^b^	0.963	0.471 ^b^	0.992	0.275 ^b^	0.960
M5-N_2_	0.527 ^b^	0.992	0.396 ^b^	0.976	0.459 ^b^	0.990	0.298 ^b^	0.973
M6-Air	0.643 ^a^	0.994	0.505 ^a^	0.966	0.556 ^a^	0.985	0.413 ^a^	0.976
M6-Ar	0.467 ^bc^	0.994	0.390 ^b^	0.987	0.436 ^b^	0.986	0.274 ^b^	0.966
M6-N_2_	0.483 ^b^	0.992	0.415 ^b^	0.992	0.437 ^b^	0.992	0.298 ^b^	0.970

Different letters in the column indicate a statistically different value (*p* ≤ 0.05).

## Data Availability

Data is contained within the article or Supplementary Materials.

## Supplementary Materials

The following supporting information can be downloaded at: https://www.mdpi.com/article/10.3390/foods12142672/s1, Figure S1: Strip-plot design of the experimental field in the two years. P arranged in vertical strips as the main plot (P1 = 46 kg/ha, P2 = 98 kg/ha), N was assigned to the vertical sub-plots (N1 = 45 kg/ha, N2 = 90 kg/ha, N3 = 135 kg/ha), and varieties were applied horizontally in sub-sub-plots (Bologna, Bolero, Pandas, Verna) with three replicate blocks per year. Each sub-sub-subplot was 1800 m^2^; Figure S2: Linear regression of the decrease of weight (%): (a) year 2019; (b) year 2020; Figure S3: Linear regression of the decrease of water activity (%): (a) year 2019; (b) year 2020; Figure S4: Hedonic index (HI) of the bread after baking (t = 0): (a) year 2019; (b) year 2020. The red line indicates the HI reference limit of shelf-life. Different lowercase letters indicate significant differences at *p* < 0.05; Table S1. Average of the chemical and technological parameters for the 6 treatments (T) and for the years 2019 and 2020 (Y).
