# Supplementary material for: Effect of Fertilization Regime of Common Wheat (Triticum aestivum) on Flour Quality and Shelf-Life of PDO Tuscan Bread
Source: Foods. 2023 Jul 11;12(14):2672. doi: 10.3390/foods12142672 (PMC10379306; doi:10.3390/foods12142672)
Supplement: Supplementary file 1 [file foods-12-02672-s001.zip › foods-2487151-supplementary.pdf]

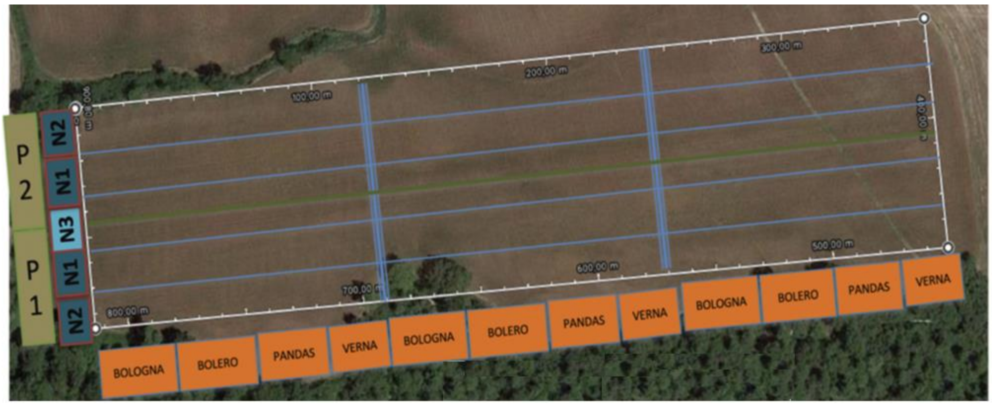

Figure S1. Strip-plot design of experimental field in the two years. P arranged in vertical strips as the main plot (P1=46 kg/ha, P2=98 kg/ha), N was assigned to the vertical sub-plots (N1=45 kg/ha, N2=90 kg/ha, N3=135 kg/ha), and varieties were applied horizontally in sub-sub-plots (Bologna, Bolero, Pandas, Verna) with three replicate blocks per year .Each sub-sub-subplot was 1800 m^2.

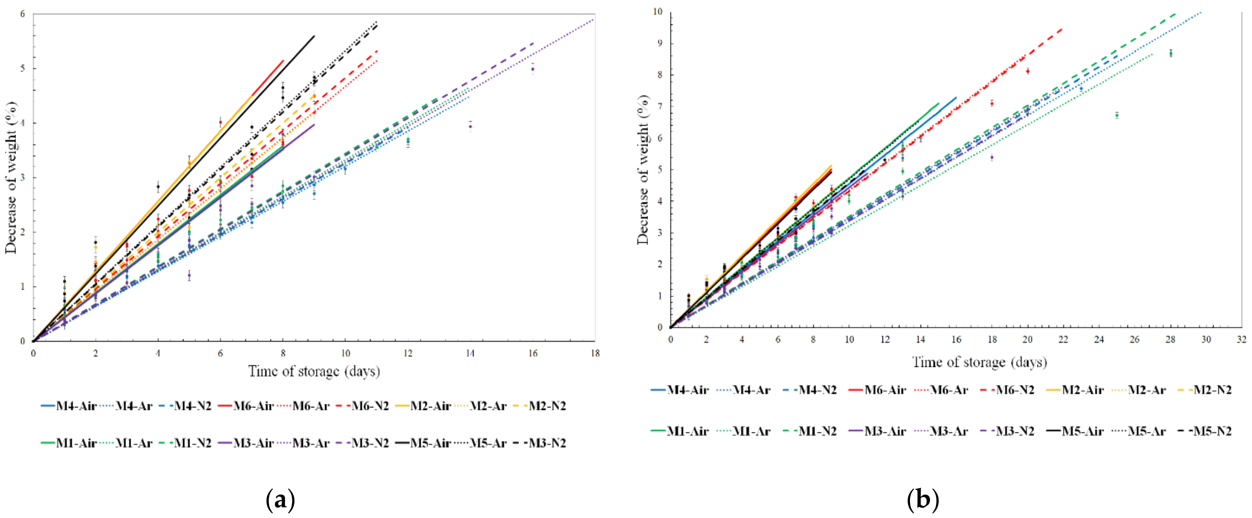

Figure S2. Linear regression of the decrease of weight (%): (**a**) Year 2019; (**b**) Year 2020.

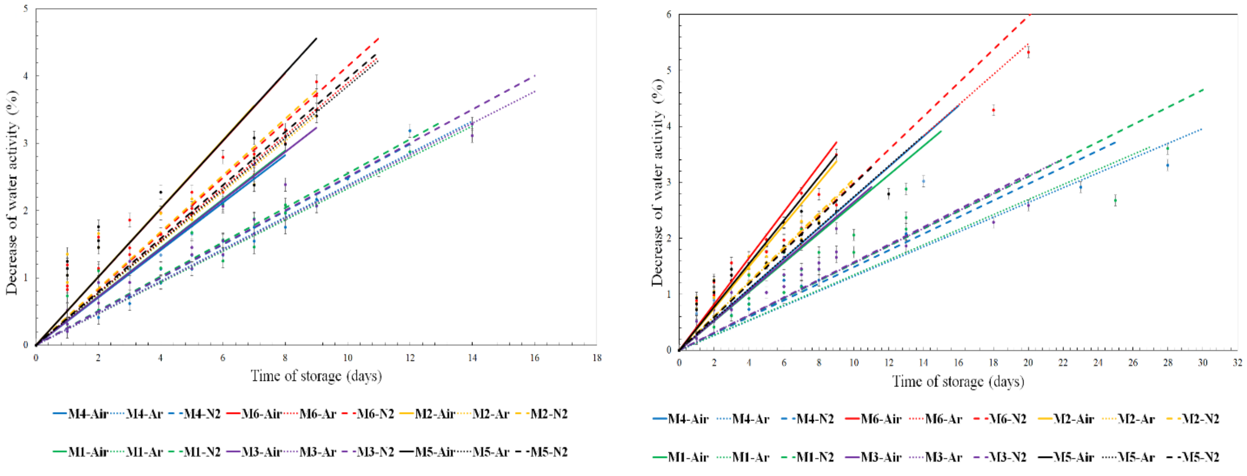

(**a**) (**b**)

Figure S3. Linear regression of the decrease of water activity (%): (**a**) Year 2019; (**b**) Year 2020.

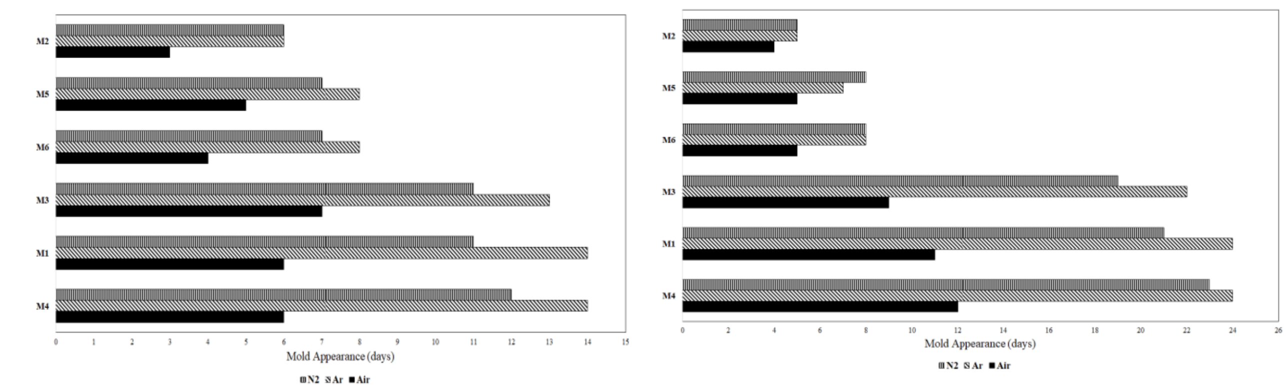

(**a**) (**b**)

Figure S4. Hedonic index (HI) of the bread after baking (t=0): (**a**) Year 2019; (**b**) Year 2020. The red line indicates the HI reference limit of shelf-life. Different lowercase letters indicate significant differences at $p < 0.05$.

Table S1. Average of the chemical and technological parameters for the 6 treatments (T) and for the years 2019 and 2020 (Y).

		Average										
**Parameters**	**Units**	**M1**	**M2**	**M3**	**M4**	**M5**	**M6**	**(T)[1]**	**2019**	**2020**	**(Y)[1]**	**(T × Y)[1]**
**Chemical**												
Humidity	% w/w	11.11	11.07	11.07	10.95	10.55	11.02	ns	10.87	11.06	ns	ns
Ashes	% w/w	1.29	1.27	1.31	1.23	1.20	1.23	ns	1.16^{b}	1.35^{a}	***	ns
Proteins	% w/w	12.79^{c}	12.97^{c}	13.17^{bc}	13.44^{ab}	13.86^{ab}	14.01^{a}	**	13.87^{a}	12.88^{b}	***	ns
Total fats	% w/w	2.25	2.35	2.29	2.24	2.26	2.30	ns	2.10	2.47	ns	ns
Total dietary fiber	% w/w	5.42^{c}	7.83^{a}	5.98^{c}	6.01^{c}	6.72^{b}	6.81^{b}	***	5.81^{b}	7.11^{a}	***	ns
Sucrose	% w/w	0.87^{b}	0.74^{d}	0.92^{a}	0.91^{a}	0.81^{c}	0.80^{c}	***	0.77^{b}	0.91^{a}	***	***
Glucose	% w/w	0.37^{b}	0.27^{c}	0.38^{a}	0.39^{a}	0.30^{c}	0.31^{c}	***	0.27^{b}	0.40^{a}	***	ns
Fructose	% w/w	0.12	0.11	0.12	0.13	0.12	0.13	ns	0.11	0.13	ns	ns
Maltose	% w/w	6.12^{c}	5.47^{f}	6.21^{b}	6.28^{a}	5.83^{d}	5.70^{e}	***	4.92^{b}	6.95^{a}	***	***
Wet gluten	% w/w	39.22^{c}	36.17^{d}	39.15^{c}	38.64^{c}	41.22^{b}	42.36^{a}	**	39.11	39.80	ns	ns
Dry gluten	% w/w	12.01^{c}	11.03^{d}	12.04^{c}	12.30^{bc}	12.44^{b}	13.34^{a}	***	11.66^{b}	12.73^{a}	**	**
Gluten index	% w/w	67.62^{c}	66.21^{c}	72.23^{a}	71.32^{ab}	70.63^{b}	72.92^{a}	*	68.32^{b}	71.98^{a}	*	ns
Total Starch	% w/w	84.97^{c}	84.02^{d}	85.75^{b}	85.37^{bc}	86.83^{a}	87.02^{a}	***	84.34^{b}	86.92^{a}	***	ns
Amylose	% w/w	21.77^{b}	24.14^{a}	21.78^{b}	21.84^{b}	24.73^{a}	24.42^{a}	***	24.00^{a}	22.22^{b}	***	ns
Amylopectin	% w/w	78.26^{a}	75.94^{b}	78.27^{a}	78.25 ^{a}	75.32^{b}	75.68^{b}	***	76.07^{b}	77.83^{a}	***	ns
Falling number	seconds	318	315	320	319	316	321	ns	307	329	ns	ns
Total polyphenol	mg GAE/kg dm	735^{a}	599^{b}	722^{a}	742^{a}	580^{b}	566^{b}	***	533^{b}	781^{a}	***	**
Total flavonoids	mg CE/kg dm	65.82^{b}	54.93^{d}	63.45^{c}	67.42^{a}	50.44^{f}	53.42^{e}	***	50.33^{b}	68.16^{a}	***	***
ABTS	µmol TE/g dm	0.97^{ab}	0.68^{c}	0.89^{b}	1.03^{a}	0.65^{c}	0.54^{d}	***	0.62^{b}	0.96^{a}	***	ns
DPPH	µmol TE/g dm	0.55^{ab}	0.42^{c}	0.52^{b}	0.60^{a}	0.41^{c}	0.40^{c}	***	0.37^{b}	0.60^{a}	***	**
FRAP	µmol TE/g dm	1.15^{ab}	0.89^{c}	1.11^{b}	1.19^{a}	0.86c	0.74^{d}	**	0.67^{b}	1.31^{a}	***	ns
**Technological**												
W	10^{-4} joules	239	255	248	250	266	272	ns	246	263	ns	ns
P/L		2.12^{b}	2.42^{a}	2.04^{b}	2.17^{b}	2.48^{a}	2.56^{a}	**	1.80^{b}	2.80^{a}	*	ns
P	mm	131	137	128	132	136	137	ns	110^{b}	157^{a}	**	ns
L	mm	56^{a}	48^{b}	55^{a}	58^{a}	46^{b}	48^{b}	*	44^{b}	59^{a}	*	ns
G		14.5^{a}	13.4^{b}	14.6^{a}	14.4^{a}	13.7^{b}	13.8^{b}	*	13.1^{b}	15.0^{a}	*	ns
Water absorption	%	67.9^{b}	71.4^{a}	67.9^{b}	67.7^{b}	71.0^{a}	70.5^{a}	***	69.0	69.7	ns	*
Dough time	Minutes	5.1	4.7	5.3	4.8	5.0	4.8	ns	5.4	4.4	ns	ns
Stability	Minutes	5.6	5.1	5.1	5.4	5.5	5.6	ns	4.8	5.9	ns	ns
E10	UF	44	54	50	48	53	47	ns	47	51	ns	ns
E(ICC)	UF	78	82	78	85	83	84	ns	76	86	ns	ns
FQN		80	69	75	77	71	70	ns	69	78	ns	ns

[1] Significance level: *** $p \le 0.001$; ** $p \le 0.01$; * $p \le 0.05$; ns=not significant ($p > 0.05$). In the same row, different letters indicate significant differences among samples.
